# Assessment of the Practices and Perspectives of Healthcare Providers Towards Utilization of Telemedicine for the Care of Adult Patients With Diabetes Mellitus During the COVID-19 Pandemic in India

**DOI:** 10.7759/cureus.54735

**Published:** 2024-02-22

**Authors:** Shubham Atal, Sowrabha Bhat, Sayan Kumar Das, Rajnish Joshi, Aditi Pandit Kabde, Aishwarya Krishnamurthy, Tejal Lathia, Balakrishnan Sadasivam, Rukiya Surya Shaikh

**Affiliations:** 1 Pharmacology, All India Institute of Medical Sciences, Bhopal, Bhopal, IND; 2 Endocrinology, Diabetes and Metabolism, Yenepoya Medical College Hospital, Mangalore, IND; 3 Pharmacology, Manipal Tata Medical College, Jamshedpur, IND; 4 Pharmacology, Manipal Academy of Higher Education, Manipal, IND; 5 Internal Medicine, All India Institute of Medical Sciences, Bhopal, Bhopal, IND; 6 Endocrinology, Narayana Superspeciality Hospital, Nanded, IND; 7 Endocrinology, Max Superspecialty Hospital, Ghaziabad, IND; 8 Endocrinology, Apollo and Cloud Nine Hospitals, Navi Mumbai, IND; 9 Endocrinology, Blue Circle Clinic, Mumbai, IND

**Keywords:** diabetes, telehealth, ehealth, telecare, telediabetes

## Abstract

Background and purpose

The lockdowns and restrictions enforced periodically during the COVID-19 pandemic posed a serious challenge for non-COVID care, especially in diabetes where telediabetes, the utilization of telemedicine consultations for diabetic care, became more necessary than ever before. Although studies have shed light on the perception of patients, there is a paucity of studies from the perspective of healthcare providers, especially in an Indian context. Moving forward, it is imperative to understand the perspectives of telediabetes providers in this domain. Hence, a nationwide survey was carried out to assess providers' practices and perspectives towards using telemedicine for providing diabetes care in India during the COVID-19 pandemic and beyond.

Methods

An online questionnaire-based, cross-sectional study was carried out involving diabetes care physicians. The study tool was developed after the identification of broad themes and constructs from published literature, national guidelines, and diabetes experts’ recommendations, following which, it was validated by six experts and pilot-tested. An online open survey, hosted on a professional platform, was circulated to internists, endocrinologists, and other diabetes care physicians of various institutions, hospitals, and clinics from both public and private sectors across the country through individual and group emails and various mobile messenger services.

Results

Out of the 239 doctors who responded to the survey, 195 (81.6%) had provided telediabetes services since the COVID-19 outbreak, and 84.1% were actively providing teleconsultations for diabetes at the time of the survey. The majority of participants (63.2%) were private practitioners. Telediabetes engagement was 3.5 hours per day at the peak of the pandemic and reduced significantly to one hour after the end of the pandemic. Video calling was the most preferred modality for consultation, whereas messaging services were preferred for input from the patients. Printed prescription images followed by text messages were the common modalities for sending treatment advice. The overall perception towards telediabetes was positive (50.1%). Most physicians reported being reasonably and somewhat aware (65.6% and 20.5%, respectively) of telemedicine practice guidelines but were not sure about the extent of compliance.

Conclusions

Our study sheds light not only on the utilization of telediabetes from physicians’ perspectives and practices but also on its acceptability while identifying areas requiring clarity and focus moving forward.

## Introduction

Individuals with diabetes remained at an increased risk of developing serious COVID-19 and its complications due to multiple reasons, with up to 50% higher mortality rates reported. The complete or partial ‘lockdowns’ and restrictions enforced periodically due to this pandemic affected people with diabetes on various fronts such as adherence to diet, exercise, availability of medicines or investigations, and additional mental stress [1]. During the COVID lockdowns in 2020 and 2021, serious challenges were posed in India for non-COVID care, with patients’ movements being restricted along with the closure of routine outpatient clinics in many centres, which were designated as COVID care hospitals from time to time. Apprehensions and fear of contracting COVID among patients and care providers also saw footfalls in hospitals and clinics, which went down significantly. In such a scenario, providing in-person consultations, especially follow-ups, proved difficult for many diabetes care providers. The alternative approach that was adopted by many diabetes physicians is telemedicine consultations through video/audio modes, that is, telediabetes. Notification of the National Medical Council’s Guidelines for Practice of Telemedicine in India provided clarity and structure for such consultations [2]. Effective telemedicine strategies and their general acceptability are still evolving in the country. The Research Society for the Study of Diabetes in India (RSSDI) clinical practice recommendations for the management of type 2 diabetes, released in 2022 [3], stress the application of telediabetes for encompassing all aspects of outpatient care in dysglycemia among individuals with diabetes, with an aim of utilizing remote consultations as a mode of primary prevention against COVID-19 infections.

In light of the pandemic, telemedicine has not only bridged the gaps in accessibility to healthcare but has also shown the potential to become the central piece in the delivery of healthcare in the future [4,5]. From the patients’ perspective, telemedicine may offer convenience and lower cost, possibly relegating in-person visits to a secondary option for addressing their needs [6]. It is equally important to understand the providers’ perspectives towards teleconsultations for diabetes, to know where we stand presently in terms of telediabetes in India, its future scope, and ways to improve it. While no Indian studies have specifically addressed this, a review was conducted in the U.S. to examine the utilization of telemedicine amidst COVID-19 and to evaluate the benefits of continuing its usage in the future. Aside from the various benefits of telemedicine, it pointed out disadvantages such as the lack of available technological resources, issues with data security, and challenges in performing traditional patient examinations. It pointed out changes that can help integrate telemedicine services more efficiently into the healthcare landscape, in preparedness for future pandemics, as well as generally [7].

While telediabetes has become more popular and necessary than ever before with the COVID-19 pandemic, it has its own strengths and limitations. Going forward through this pandemic and beyond, it is very important to understand both patients’ and providers’ perspectives in this domain. We carried out a nationwide survey to assess providers' practices and perspectives towards using telemedicine for providing diabetes care during the COVID-19 pandemic in India.

## Materials and methods

This questionnaire-based cross-sectional study was carried out across three months at the beginning of 2022 after obtaining approval from the Institutional Human Ethics Committee (Approval No.: IHEC-LOP/2021/IL001), All India Institute of Medical Sciences, Bhopal, India. The survey was designed after the identification of broad themes and constructs regarding physicians' perspectives towards using telemedicine for diabetes from published literature, national guidelines, and expert recommendations regarding telediabetes. This was done in conjunction with thorough discussion within the study team, which consisted of diabetes experts. The identified domains finally included physicians’ demographic and professional characteristics, nature of engagement, beneficiaries, perceptions, and future scope.

Grounded in evidence, items were then constructed, which were representative of the constructs of each domain. Expert opinion was sought where each expert was asked to rate each item using a Likert scale bearing three levels with ‘1’ being ‘not necessary’, ‘2’ being ‘useful’, and ‘3’ being ‘essential’, and a weighted value was assigned to each rating. A total of six independent expert opinions were sought, following which the responses were pooled. The number of responses, rating an item as ‘essential’, was determined. As there was a lack of consensus on certain items among the experts, the content validation ratio (CVR) was calculated for each item using the formula: 

CVR = {(n)-(N/2)}/(N/2) 

where n = Total number of experts rating an item as ‘essential’; N = Total number of experts

**Figure 1:**
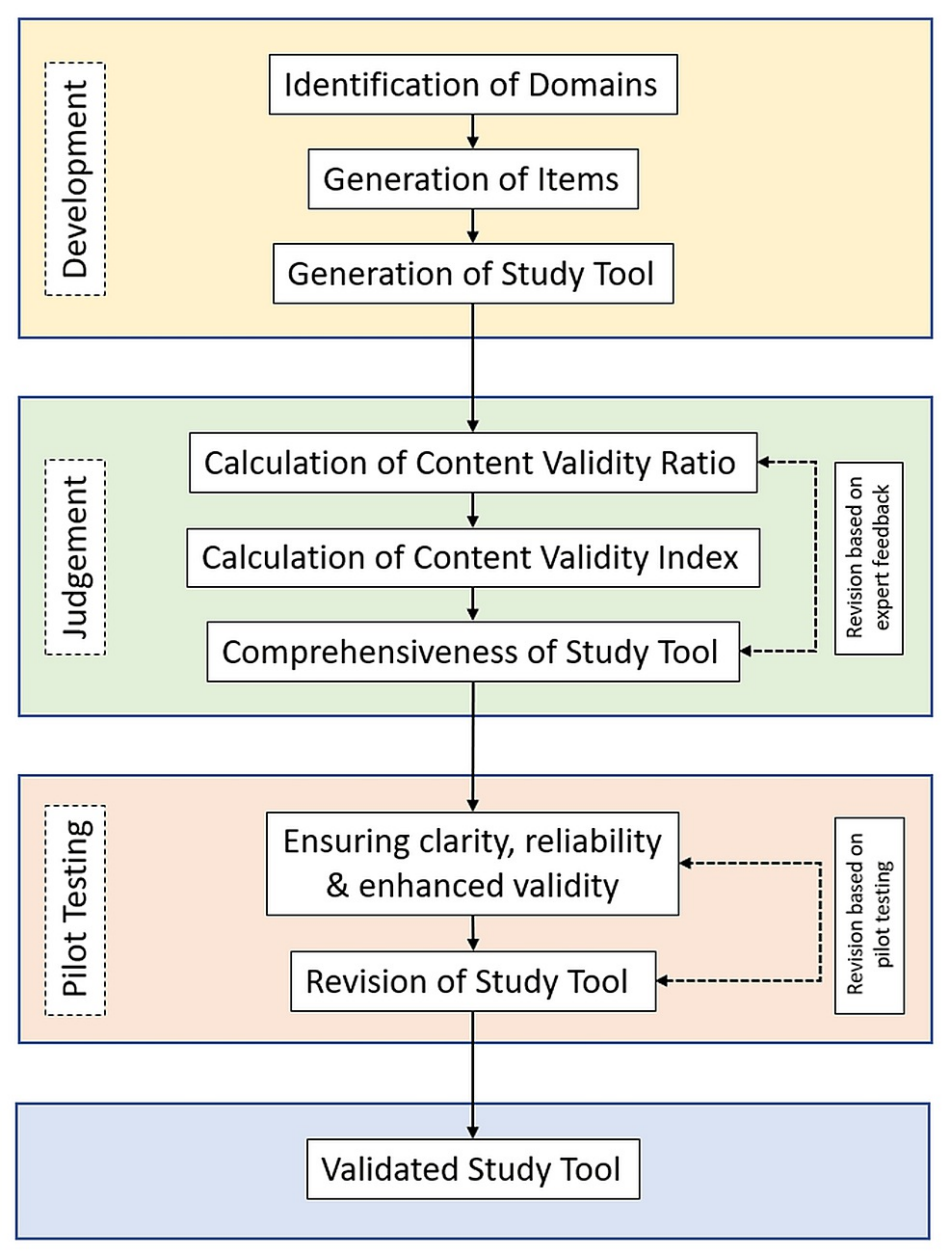
Flow diagram of the process of development and validation of the study tool

A CVR of more than 0.78 was considered evidence of good content validity and was included in the final questionnaire. The content validity index (CVI), which is the mean of all the CVR values calculated for every item, for the questionnaire, was calculated and was seen to be 0.99. The final developed study tool was pilot-tested in a small number of participants who were representative of the study population for identifying ambiguity, ensuring clarity and reliability. The process of development and validation of the study tool is outlined in Figure 1.

As the study was designed to be exploratory in nature, convenience sampling was used where internists, endocrinologists, and other diabetes care physicians of academic/non-academic institutions, hospitals, and clinics from both public and private sectors across the country were invited to participate in the online open survey, hosted on a professional platform, circulated individually and within groups, through emails and various mobile messenger services such as WhatsApp. This was done due to the absence of a country-wise database of physicians who were providing telediabetes consultations. As the study group consisted of investigators from various regions of the country, an effort was made to have equal participation from different regions of the country. Informed consent was obtained from participants before proceeding to answer the survey and no incentives were offered for participating in the survey. Data were transferred onto Microsoft Excel to create the study database, and EZR (Easy R), R Commander, version 2.4-0) [8] was used for all statistical calculations. Descriptive statistics were supplemented with appropriate graphical representations to display the results.

## Results

A total of 239 doctors responded to the online study survey. Their demographic and professional characteristics are displayed in Table 1. The mean age of the respondents was 42.6 (± 9.6) years, with 63.6 % males. More than half the participants possessed a super specialization in endocrinology, with a further 28.5% having a specialization in general medicine. The average experience of the respondents was around 14.5 years. The majority of the doctors (87%) practiced in urban areas, and around one-third of the participants were from southern and western India.

**Table 1:** Demographic and professional characteristics of respondents

Characteristic		Frequency (%) / Mean N = 239
Mean Age (±SD) in years		42.6 (± 9.6)
Gender	Male	152 (63.6%)
	Female	82 (34.3)
	Not Answered	5 (2.1%)
Professional Qualification	DM/DNB Endocrinology	123 (51.5%)
	MD/DNB General Medicine	68 (28.5%)
	Diploma/Fellowship endocrinology or diabetes	16 (6.7%)
	MRCP	04 (1.7%)
	MBBS	15 (6.3%)
	Others (PhD, BAMS)	13 (5.3%)
Years of Experience	Mean (±SD)	14.5 (± 8.7)
	Minimum	1
	Maximum	42
Geographical region of practice (in India)	North	27 (11.3%)
	South	73 (30.5%)
	East	28 (11.7%)
	West	81 (33.9%)
	Central	30 (12.6%)
Location of Practice	Urban	208 (87%)
	Semi-Urban	27 (11.3%)
	Semi-Rural	01 (0.4%)
	Rural	03 (1.3%)
Provided tele-diabetes services since COVID-19 outbreak	Yes	195 (81.6 %)
	No	44 (18.4 %)

Among them, 195 (81.6%) participants affirmed to have provided telediabetes services since the COVID-19 outbreak in India. The nature and pattern of telediabetes engagement of these doctors are shown in Table 2. The majority of them (84.1%) were actively providing teleconsultations for diabetes at the time of responding to the survey. However, only 40% of them had provided telediabetes prior to the outbreak of COVID-19. The majority of the respondents had their practice set up in the private sector (63.2%); only 10% of respondents were exclusively in public practice. At the peak of the COVID-19 outbreak (during the 2021 ‘wave’ in India), respondents engaged in telediabetes for around 3.5 hours per day, which came down to around one hour daily after the ease of the wave. The most preferred modality of providing consultations was video calling (39, 35.38%), followed closely by messaging (55, 28.21%) and voice calling (52, 26.67%), whereas the most common method used for receiving inputs from patients was through messaging (71.1%). The doctors used printed prescription images (46%), text messages with instructions (38.1%), and handwritten prescription images (35.1%) as the common modalities for sending treatment advice to their patients. Among the platforms used for providing telediabetes consultations, WhatsApp (70.9%) was the most commonly used public platform, followed by telephones, SMS, and Zoom. Among dedicated platforms, Healthplix (21.3%) and the hospital’s own unique app (17.6%) were the commonly used ones. Appointments were scheduled for most doctors either by themselves (41%) or by their receptionist (37.7%), and various kinds of modes were used for data record keeping. Table 2 presents the reported duration and modes of communication in telediabetes engagement of the respondents.

**Table 2:** Telediabetes engagement of respondents

Question	Response	Frequency (%) / Mean (N = 195)				
Currently Providing Tele-DM	Yes	164 (84.1%)				
	No	23 (11.8%)				
	Not answered	89 (4.1%)				
Provided prior to COVID-19 Outbreak	Yes	77 (39.5%)				
	No	118 (60.5%)				
Type of Practice	Public	24 (10%)				
	Private	151 (63.2%)				
	Both	64 (26.8%)				
Hours per day during the peak of COVID-19	Mean (± SD)	3.44 (± 5)				
Hours per day after the easing of COVID-19	Mean (± SD)	1.15 (± 0.9)				
Tele-medicine modality of engagement	Ranking (preference) - overall	#1	#2	#3	#4	#5
	Voice call	52	38	28	25	30
	Video call	69	23	22	23	37
	Message	55	19	43	26	35
	Live chat	26	17	17	25	45
	Others	20	01	01	04	49
	Others	E-mail		05		
		App		13		
Modality for receiving prescriptions/reports	Messages	170 (71.1%)				
	E-mail	66 (27.6%)				
	Entered Directly	57 (23.8%)				
	Live chat	21 (8.8%)				
Modality for sending treatment advice	Printed prescription images	110 (46%)				
	Text message with instructions	91 (38.1%)				
	Handwritten prescription images	84 (35.1%)				
	Links to external website	11 (4.6%)				
	Additional image files	09 (3.8%)				
	Voice files	07 (2.9%)				
	Video files	06 (2.5%)				
	Others	05 (2.1%)				
Public platforms used	WhatsApp	169 (70.7%)				
	Telephone	69 (28.9%)				
	SMS text	60 (25.1%)				
	Zoom	54 (22.6%)				
	Google Meet	25 (10.5%)				
	Microsoft Teams	16 (6.7%)				
	Webex	07 (2.9%)				
	Skype	13 (5.4%)				
	Telegram	02 (0.8%)				
	Signal	01 (0.4%)				
	Others: hospital’s own apps (most common), E-Sanjeevani (GOI’s App), Grapes HIS, DocPulse, 7Sugar, NH Care App, Medeva, Remedo	25 (10.5%)				
	None	01 (0.4%)				
Dedicated platforms	None	69 (28.9%)				
	Heathplix	51 (21.3%)				
	Unique Hospital’s Apps	42 (17.6%)				
	Docon	27 (11.3%)				
	Practo	17 (7.1%)				
	Lybrate	15 (6.3%)				
	Docplexus	10 (4.2%)				
	M-fine	06 (2.5%)				
	E-Vaidyak	01 (0.4%)				
	Others [not specified]	29 (12.1%)				
Responsible for scheduling appointments	Myself	98 (41%)				
	Receptionist	90 (37.7%)				
	Self-registration	43 (18%)				
	Colleague/associate	25 (10.5%)				
	Others: call centre, institute/hospital, no formal appointments	09 (3.8%)				
Modality for data record-keeping	Integrated software	94 (39.3%)				
	Register	50 (20.9%)				
	Excel	31 (13%)				
	Google Sheet	10 (4.2%)				
	None	45 (18.8%)				

**Figure 2:**
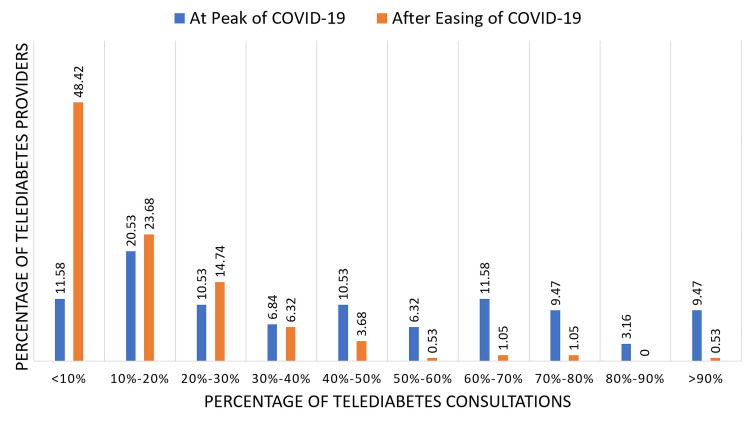
Percentage of telediabetes consultations at the peak of the COVID-19 pandemic vs after the ease of the COVID-19 pandemic

An increase in the utilization of telediabetes was observed during the peak of the COVID-19 pandemic where 60%-70% of the total consultations were via telediabetes for 11.58% of the participants, and 9.5% provided more than 90% of their total consultations as telediabetes consultations. A shift in the utilization of telediabetes was noted after the ease of the pandemic where 48.4% of participants reported the utilization of telediabetes to be less than 10% (Figure 2).

Table 3 shows the characteristics of the patients being seen in the diabetes teleconsultations. The majority of the doctors reported that their telediabetes beneficiaries are a mix of both new and follow-up cases (59.5%), which are patients consulting for the first time and patients coming for subsequent consultations and males and females (82.1%). Patients are more commonly middle-aged adults, ages 31-59 years (56.9%). Most doctors, in their teleconsultations, see patients who are previously registered (76.4%); walk-ins (32.8%), that is, patients asking for telediabetes consultations; and referrals from other doctors (28.2%). In terms of the locality from which patients come, it is usually a mix of local and other vicinities (62.6%), whereas the socioeconomic strata of the patients were reported almost equally as mixed or middle-income strata.

**Table 3:** Characteristics of telediabetes beneficiaries (patients)

Characteristics		Frequency (%), N = 195
Main telediabetes beneficiaries	Mix of both follow-up and new patients	116 (59.5%)
	Follow-up patients	73 (37.4%)
	New patients	01 (0.5%)
	Not answered	05 (2.6%)
Modalities of identification and/or invitation	Previously registered	149 (76.4%)
	Walk-in (patients asking for telediabetes consultations themselves)	64 (32.8%)
	Referral from other doctors	55 (28.2%)
	Advertisement of tele-diabetes	30 (15.4%)
	Through commercial service	11 (5.6%)
	Others	07 (3.6%)
Age group	Middle-aged adults (31 to 59 years)	111 (56.9%)
	Young adults (17 to 30 years)	18 (9.2%)
	Elderly (above 60 years)	14 (7.1%)
	Children (below 16 years of age)	00 (0%)
	Not specific	52 (26.7%)
Gender	Mixed population (both men and women)	160 (82.1%)
	Women	16 (8.2%)
	Men	15 (7.7%)
	Not sure	04 (2.01%)
Locality/geographical area	Local area/vicinity	44 (22.6%)
	Other cities/towns	26 (13.3%)
	Mixed population (both local and from other cities)	122 (62.6%)
	Not sure	03 (1.5%)
Socioeconomic strata	Upper	24 (12.3%)
	Middle	82 (42.1%)
	Lower	01 (0.5%)
	Mixed population (from all socioeconomic strata)	84 (43.1%)
	Not sure	04 (2.1%)

Easier accessibility was considered the chief advantage of the use of teleconsultations for diabetes by the majority of the doctors (88.7%), whereas difficulty in diagnosis due to the lack of physical examination (92.3%) and difficulty in the management of acute complications (72.3%) were perceived as the major disadvantages. A considerable proportion of respondents also felt that telediabetes improved the effectiveness of therapeutic interventions (51.8%) and allowed the provision of psychological support to patients (46.2%), while the management of elderly/pregnant/multiple comorbidities was found difficult (50.8%). The overall perception towards telediabetes was either positive (50.1%) or neutral (30.3%) among the majority of the doctors whereas 10% had a very positive perception. When it comes to awareness regarding recommendations or provisions related to telemedicine, most of the doctors reported being reasonably or somewhat aware (65.6% and 20.5%, respectively). However, they were not as sure about the extent of compliance with such recommendations; 44.1% responded saying they were partially compliant, whereas 22.1% did not know if they were. More than 70% of doctors agreed that telediabetes will grow in India in the coming future, either expanding greatly or progressing slowly. The question on improvement of telediabetes drew variable responses - patient awareness and education (72.8%) and more clarity regarding medico-legal aspects of telediabetes (59.5%) were thought to be the most important aspects to focus on for improving the effectiveness of telediabetes services. Finally, 60% of doctors were likely to continue telediabetes services after the pandemic, which is reflected by their response with a score of 6 or more out of 10. Most notably 19% of the doctors indicated a full score of 10. Table 4 presents the providers’ perspectives towards telediabetes and its future scope.

**Table 4:** Providers’ perceptions of telediabetes and its future scope

Questions	Responses	Frequency (%) (N = 195)
Advantages of providing telediabetes consultations?	Easier accessibility	173 (88.7%)
	Improved effectiveness of therapeutic intervention	101 (51.8%)
	Psychological support for the patient	90 (46.2%)
	Improvement of compliance	89 (45.6%)
	Enhancement of quality of care	44 (22.6%)
	Others - Economic benefit – Minimizes cost of travelling - Time saved – Travelling time and waiting time - Easy access for bedridden patients - Social distancing - Improved follow-up in even non-working hours - Better communication due to patients being in their own environment	10 (5.1%)
Disadvantages of providing telediabetes consultations?	Difficulty in diagnosis due to lack of physical examination	180 (92.3%)
	Difficulty in the management of acute complications	141 (72.3%)
	Difficulty in managing elderly/pregnant women/multiple comorbidities	99 (50.8%)
	Inconvenient and time-consuming	78 (40%)
	Limitations in drug prescribing	49 (25.1%)
	Issues related to monetary compensation	35 (17.9%)
	Unwanted change in trends of practice	22 (11.3%)
	High infrastructural investment	09 (4.6%)
	Others: - Medicolegal issues - Prescribing restrictions due to government regulations - Lack of proactive follow-up from the patient’s end - Absence of emotions shared	05 (2.6%)
Overall perception	Strongly negative	02 (1%)
	Negative	16 (8.2%)
	Neutral	59 (30.3%)
	Positive	98 (50.1%)
	Strongly positive	20 (10.3%)
Awareness related to provisions/recommendations related to telediabetes	Yes	128 (65.6%)
	No	27 (13.8%)
	Somewhat	40 (20.5%)
The extent of compliance with the provisions/recommendations in the guidelines?	Completely	66 (33.8%)
	Partially	86 (44.1%)
	Don’t know	43 (22.1%)
What will be the future of telediabetes in India?	Will expand greatly in the next few years	76 (39%)
	Will grow slowly over the next few years	66 (33.8%)
	Will remain more or less at the same level as now in the next few years	35 (17.9%)
	Providers will revert back to usual face-to-face consultations once the pandemic is over	18 (9.2%)
How can tele-diabetes be improved and made more effective?	Patient awareness and education	142 (72.8%)
	Provider training	85 (43.6%)
	Development of dedicated platforms	106 (54.4%)
	Modification of guidelines	83 (42.6%)
	Improvement in Internet connectivity	71 (36.4%)
	More clarity regarding medico-legal aspects	116 (59.5%)
	Others: Should be discouraged, telemedicine increases the chance of negligence and non-compliance	02 (1%)
	Not Answered	08 (4.1%)
How likely are you to continue telediabetes services after the pandemic?	1	10 (5.1%)
	2	06 (3.1%)
	3	18 (9.2%)
	4	15 (7.7%)
	5	29 (14.9%)
	6	13 (6.7%)
	7	32 (16.4%)
	8	26 (13.3%)
	9	09 (4.6%)
	10	37 (19%)

## Discussion

In this online survey to assess physicians' perceptions towards the use of telemedicine in diabetes, the majority of the 239 respondents (81.6 %) provided telediabetes consultations since the COVID-19 outbreak and had a super-specialist degree in endocrinology (63.5%), with an average experience of 14.5 years. This is reflective of the fact that participation in the study was from physicians primarily involved in diabetes care. Interestingly, only 40% of these doctors had practiced telediabetes prior to the pandemic, reflecting a change in practice patterns in a chronic non-communicable disease such as diabetes. An urban setting makes for easier access to modern amenities such as healthcare infrastructure and internet connectivity, which form the backbone for the provision of telediabetes care [8]; accordingly, we received 87% of responses from doctors practicing in urban areas. In a similar cross-sectional study conducted in 2021, involving 83 DO and MD physicians, including residents treating patients with type 2 diabetes mellitus in Florida, USA, the majority of participants were from the specialty of family medicine (n=47) and internal medicine (n=21) and very few endocrinologists, with a mean age 56 ± 10.5 years [9]. It was also seen that more than a quarter of care criteria surged from 6% to 37.3% pre- to post-COVID in telehealth.

The study calculated some unique matrices previously not included in similar surveys. The average duration of telediabetes engagement was much higher at 3.5 hours per day at the peak of the COVID-19 wave in mid-2021, compared to one hour after the ease of the wave, attributable to the stringent restrictive measures implemented during the wave, which made accessing healthcare facilities difficult along with apprehension among the public. The conversion of many healthcare facilities into specialized COVID care centers to tackle the urgent burden of the pandemic further accentuated these issues during the peak pandemic months [10,11]. Video calling, voice calling, and messaging were the modalities of communication used for delivering the teleconsultations in order of preference. The ease of video calling using phone-based applications has definitely aided in providing easier access to telediabetes, but inaccessibility or unstable internet connectivity acts as a hindrance. Alternatively, voice calling and text-based messaging are relatively easily accessible modalities, which aid in filling these voids.

Prescription documentation forms the backbone of any healthcare consultation as the prescription is the key transaction between the physicians and the patients [12]. In our study, images of printed and hand-written prescriptions (46% and 35.1%, respectively) were the most commonly used modality of sharing medical advice, followed by text messages - all modalities being convenient for the patient. Being a legal document [12], proper storage of the prescription in a tangible medium is imperative on behalf of both the physician and the patient. As per the national telemedicine guidelines issued by the government in India, maintenance of a digital trail and documentation of the consultation is mandatory from the physician's perspective, the same as that of in-person consultations [2]. From the patients' perspective, the prescription not only enumerates the medicines prescribed but also acts as the reference for future consultations detailing treatment history and the course of the disease.

In our study, WhatsApp (70.9%) was the preferred mobile application among public platforms, and Healthplix (21.3%) and then the hospital's unique app (17.6%) were the dedicated platforms that saw the most use for telediabetes consultations. With over 487 million users in India [13], WhatsApp, with its ease of use and accessibility, is one of the most commonly used mobile applications, allowing not only video calling but also the sharing of media files. The survey was able to assess the nature of beneficiaries, being a mix of both new and follow-up cases (59.5%) and male and female patients (82.1%). Patients are commonly middle-aged adults (56.9%) hailing from both local and other vicinities (62.6%), belonging to either mixed or middle-income strata. The ease of access coupled with the convenience of minimizing conveyance at a time when movement restrictions are in place as a conscious decision for minimization of exposure to COVID-19 is likely to have played a role in expanding the acceptability of telemedicine for the management of diabetes. Numerous patient-focused studies regarding attitude perceptions towards telemedicine in general and in diabetes have been published, with largely positive perceptions in terms of satisfaction and utility, enhancing quality of life along with outlining barriers such as technical issues and the lack of personal interaction [14-17].

While quite a few studies, a systematic review and meta-analyses, have addressed physician perceptions towards telemedicine in general pre- and post-COVID, those addressing its use in diabetes are relatively few. The effectiveness of telediabetes modality has been proven through multiple studies. In a pre-COVID meta-analysis of 13 randomized controlled trials, telemedicine was associated with a statistically significant and clinically relevant reduction in HbA1c level but did not show such an effect on blood pressure and LDL cholesterol reduction [18]. In a systematic review of 37 studies to assess physician satisfaction with telemedicine, which included varied types of telemedicine or provider teleconsultation and different diagnoses or care situations, it was shown that physicians across specialties, geographic locations, practice locations, and care situations appear satisfied with telemedicine.

We observed that easier accessibility (88.7%) and improved effectiveness of therapeutic interventions and psychological support to patients were considered advantages of telediabetes consultations by the majority of the doctors. The lack of physical examinations, leading to a difficulty in diagnosis (92.3%) and difficulty in the management of acute conditions (72.3%), was perceived as disadvantages, observations which are in accordance with the findings of earlier studies, such as that conducted by Naik et al. [19] The overall perception towards telediabetes was either positive (50.1%) or neutral (30.3%). In the study by Abidi et al. [10], the main issues with telediabetes that were identified included the lack of a regular physical exam, which is perceived to be necessary to properly treat patients (78.4%) (n=65), and 48.2% did not believe that a more complete picture of the case was not available with remote consultations. In terms of satisfaction, 47.0% (n=39) were not as satisfied with remote consultations as with face-to-face patient visits. In that study, while 68.7% (n=57) physicians believed that telediabetes is necessary to adequately treat patients, 45.8% (n=38) reported concerns regarding the admission of patients for acute medical care [20]. In another study, 68 physicians participated in understanding perceptions towards the use of teleconsultations at home for T2DM patients, and doubt concerning the quality of patient examination was identified as the major barrier. The study showed that time-saving, increase in productivity, improvements in patient health and management, and cost reduction are the major drivers [21]. In a pre-COVID study conducted in 2016 on 55 Iranian physicians and nurses, factors influencing the use of telemedicine in diabetes management were identified. It was seen that the healthcare professionals had good intentions to use telemedicine in diabetes management but had concerns regarding compatibility with other clinical activities and its overall usefulness [22]. In a Romanian cross-sectional study on GPs (family physicians), 43% observed patients' satisfaction with telemedicine, while half felt patients encountered difficulties using the technology. Overall, 62.5% of physicians expressed that patients felt well treated by virtual visits [23]. Thus, telemedicine in general, including telediabetes, has aided in bridging the gap in patient-physician communication, but it is not without its own share of limitations. Telemedicine limits the observations and subtle revealing changes that play a crucial role in the diagnosis and treatment, especially in the management of acute complications. For some, the screen acts as a physical barrier hindering the trust between the patient and the healthcare provider [24].

In light of the pandemic, telediabetes and telemedicine as a whole have seen an increase in their use, and the Ministry of Health and Family Welfare (MoHFW), Government of India (GOI), has laid down 'Telemedicine Practice Guidelines' for effective and proper telemedicine practices [2]. In our survey, most of the doctors reported being reasonably and somewhat aware (65.6% and 20.5%, respectively) regarding the recommendations or provisions related to telemedicine, but the extent of compliance with such recommendations is something they were not sure about, with 44.1% claiming partial compliance and 22.1% being unaware of the extent of their compliance. Although the guidelines issued by MoHFW enumerate the Do's and Don'ts when it comes to practicing telemedicine in India, further training has been recommended for preserving the interests of both the patients and the providers while keeping in mind the legalities associated with providing such services [2].

As with any form of healthcare, the legalities and ethical considerations are paramount when it comes to telediabetes or telemedicine. Equitable access to quality healthcare with professional integrity at affordable costs, maintenance of patient privacy, confidentiality, and protection of patient's medical data are to be ensured [25,26].

The response of 70% of the doctors in our survey is indicative of the fact that telediabetes will witness further growth in the foreseeable future, especially in the Indian context. Although the current situation pertaining to telediabetes is not without its own share of shortcomings, patient awareness and education (72.8%) and more clarity regarding medico-legal aspects of telediabetes (59.5%) were two primary areas where telehealth providers deemed to focus and clarity for improving the effectiveness of telediabetes services. The majority of the doctors responded to the survey saying that they were likely (60%) and very likely (20%) to continue providing telediabetes services even after the pandemic, which is similar to responses elicited in the study by Mubaraki et al. wherein responding doctors were in favor of continuing telediabetes consultations [19].

Our study is not without its own share of limitations. The participation rate was overall poor as the number of physicians reached out to was in the thousands. The online nature of the survey with a faceless non-personal approach through mass media could be the reason, along with the lack of time and incentives. As with most online-based surveys, our study too may have some amount of selection and response bias. As the study was conducted after the weaning of the wave of the pandemic, there is a possibility of some recall bias as well.

## Conclusions

In conclusion, telediabetes cannot be an outright substitution for a clinicians’ hands-on expertise in face-to-face consultation. However, in light of the COVID-19 pandemic where access to healthcare was met with significant restrictions, telediabetes played a crucial role in aiding the management of this chronic, non-communicable disease. Going forward, telediabetes has the potential to bridge the gap in traditional healthcare infrastructure by negating barriers such as distance, transportation, caretakers' availability, and minimizing the risk of acquiring infectious diseases. Although education and awareness are warranted, especially with respect to patient understanding and ethical and legal aspects, telediabetes is considered to be a reliable modality of treatment when it comes to quality of care and providing psychological support to patients. This study, done from physicians’ perspective, sheds light not only on the utilization of telediabetes but also its acceptability among physicians while identifying limitations and areas requiring clarity or focus moving forward.

## Appendices

Study Questionnaire: Providers’ Survey for Telediabetes

Through the assessment of the practices and perspectives of healthcare providers towards utilization of telemedicine for the care of adult patients with diabetes mellitus during the COVID-19 pandemic in India, serious challenges have been posed in India for non-COVID care, with patients’ movements being restricted along with closure of routine outpatient clinics. Fear and apprehension of contracting COVID-19 among patients and care providers has also seen footfalls in hospitals and clinics, which went down significantly. An alternative approach that has been adopted by many diabetes care providers is offering consultations or monitoring through video/audio/text modes (i.e., using telehealth for providing diabetes care). The phenomenon of 'tele-diabetes' includes communication between care provider and patient via any of multiple modalities available, such as video conferencing, telephones, smartphone apps, emails, or patient portals. Effective telediabetes strategies and the general acceptability of telediabetes are still evolving in the country. It has its own strengths and limitations. While feedback from patients regarding diabetes teleconsultations is important, it is equally important to determine the diabetes care providers’ perspectives. Hence, we invite you to participate in this simple survey, which should not take more than 8-10 minutes. If you are willing to participate, then kindly enter your basic details, and proceed to answer the questions below.

*Denotes that the question is mandatory.

Age (In Years) *:

Gender *

☐ Male

☐ Female

☐ Non-binary

What is your highest professional qualification? *

☐ MBBS

☐ MD/DNB (Medicine)

☐ DM/DNB (Endocrinology)

☐ Diploma/Fellowship in Diabetology or Endocrinology

Total years of experience in treating diabetes? (Kindly mention a number denoting the years of experience.)*:

City/Town/Place you practice *:

What is the location of your place of practice? *

☐ Urban

☐ Semi-urban

☐ Rural

☐ Semi-rural

What is the type of your practice? *

☐ Public

☐ Private

☐ Both

Email (Optional):

Have you provided telediabetes services since the outbreak of COVID-19? *

☐ Yes

☐ No

1. Are you currently providing telediabetes services? *

☐ Yes

☐ No

2. Have you provided telediabetes services prior to the outbreak of COVID-19? *

☐ Yes

☐ No

3a. What is the proportion of total consultations you have provided via tele-diabetes at the peak of the COVID-19 pandemic? *

☐ < 10%

☐ 10-20%

☐ 20-30%

☐ 30-40%

☐ 40-50%

☐ 50-60%

☐ 60-70%

☐ 70-80%

☐ 80-90%

☐ >90%

3b. What is the proportion of total consultations you currently provide via tele-diabetes, when COVID-19 pandemic has eased? *

☐ < 10%

☐ 10-20 %

☐ 20-30%

☐ 30-40%

☐ 40-50%

☐ 50-60%

☐ 60-70%

☐ 70-80%

☐ 80-90%

☐ > 90%

4a. How many hours a day on average have you provided consultations via tele-diabetes at the peak of the COVID-19 pandemic? *: 

4b. How many hours a day on average have you provided consultations via tele-diabetes when the COVID-19 pandemic has eased? *:

5. Which telemedicine modality do you use for engagement? [Rank in order of preference by picking a different number for each applicable option, with 1 being most common and 5 being least common]?

☐ Voice Call

☐ Video Call

☐ Messages (Any kind including WhatsApp)

☐ Live Chat

☐ Any other; please specify:

6. Which are the methods you employ for receiving prescriptions/reports from patients before or during consultations? [You can choose multiple options] *

☐ Messages (Any kind including WhatsApp)

☐ Live Chat

☐ Values entered directly in a database/software

☐ Emails

7. Which are the output methods you use to send treatment advice at the end of your consultations? [You can choose multiple options] *

☐ Printed prescription image Handwritten prescription image

☐ Text message with all instructions Additional Image files

☐ Voice files

☐ Video files

☐ Links to external website

8. List all public platforms you use for tele-consultations. [You can choose multiple options] *

☐ Skype Zoom

☐ Google Meet Webex

☐ Microsoft Teams WhatsApp

☐ SMS Text Telephone

☐ Telegram/Signal

☐ None

9. List all dedicated platforms you use for teleconsultations? [You can choose multiple options] *

☐ Docon Docplexus

☐ Healthplix E - Vaidyak

☐ Unique hospital /clinic software Practo

☐ Lybrate M fine

☐ None

☐ Any other (please specify):

10. Who is responsible for scheduling your tele-diabetes appointments? [You can choose multiple options] *

☐ Myself

☐ Colleague/Associate

☐ Receptionist

☐ Self-registration - integrated booking system

11. Which modality do you use for data record-keeping? [You can choose multiple options] *

☐ Register

☐ Excel sheet

☐ Google sheet

☐ Integrated/other software

☐ None

12. Who are your main tele-diabetes beneficiaries? *

☐ New patients

☐ Follow-up patients

☐ Mix of both

13. How are patients identified or invited for tele-diabetes services? [You can choose multiple options] *

☐ From previously registered patients

☐ Offered to every walk-in patient

☐ Advertisement of tele-diabetes facility separately

☐ From references of other doctors

☐ From commercial service

14. Which age group of patients do you see most commonly in tele diabetes? *

☐ Mostly young adults (18-35 years)

☐ Mostly middle-aged adults (36-64 years)

☐ Mostly elderly (65 years or more)

☐ Mostly children (less than 18 years)

☐ No Specific age group

15. Which gender of patients do you generally see in tele diabetes? *

☐ Mostly men

☐ Mostly women

☐ Mixed population

☐ Not sure

16. What locality/geographical location do your tele diabetes patients generally belong to? *

☐ Mostly from local areas/vicinities

☐ Mostly from other cities/towns

☐ Mixed population

☐ Not sure

17. Which socioeconomic strata do you perceive your tele diabetes beneficiaries mainly belong to? *

☐ Upper

☐ Middle

☐ Lower

☐ Mixed population

☐ Not sure

18. According to you, what are the advantages of providing telediabetes consultations? [You can choose multiple options] *

☐ Easier accessibility

☐ Improved effectiveness of therapeutic intervention

☐ Enhancement of quality of care

☐ Improvement of compliance

☐ Psychological support for the patient

19. According to you, what are the disadvantages of providing telediabetes consultations? [You can choose multiple options] *

☐ High infrastructural investment

☐ Inconvenient and time consuming

☐ Difficulty in diagnosis due to lack of physical examination

☐ Unwanted change in trends of practice

☐ Issues related to monetary compensation

☐ Difficulty in management of acute complications

☐ Difficulty in managing elderly/pregnant women / multiple comorbidities

☐ Limitations in drugs that can be prescribed

20. What is your overall perception of telemedicine consultations? *

☐ Strongly negative

☐ Negative

☐ Neutral

☐ Positive

☐ Strongly positive

21. Are you aware of the provisions/recommendations related to tele diabetes in the telemedicine guidelines of India and ICMR expert group guidelines? *

☐ Yes

☐ No

☐ Somewhat

22. To what extent do you comply with the provisions/recommendations in these guidelines? *

☐ Completely

☐ Partially

☐ Don't know

23. What do you feel will be the future of tele-diabetes in India? *

☐ Will expand greatly in the next few years

☐ Will grow slowly over the next few years

☐ Will remain more or less at the same level as now in the next few years

☐ Providers will revert back to usual face-to-face consultations once the pandemic is over

24. How can tele-diabetes be improved and made more effective? [You can choose multiple options] *

☐ Patient awareness and education

☐ Provider training

☐ Development of dedicated platforms

☐ Modification of guidelines

☐ Improvement in Internet connectivity

☐ More clarity regarding medico-legal aspects

25. On a scale of 1 to 10, how likely are you to continue providing tele-diabetes consultations once the pandemic is over? *

☐ 1 (Least) ☐ 2 ☐ 3 ☐ 4 ☐ 5 ☐ 6 ☐ 7 ☐ 8 ☐ 9 ☐ 10 (Most)

For any queries, please contact Dr. Shubham Atal (Principal Investigator), All India Institute of Medical Sciences, Bhopal at shubham.pharm@aiimsbhopal.edu.in.
